# Efficacy and safety of sprifermin injection for knee osteoarthritis treatment: a meta-analysis

**DOI:** 10.1186/s13075-021-02488-w

**Published:** 2021-04-09

**Authors:** Ni Zeng, Xin-Yuan Chen, Zhi-Peng Yan, Jie-Ting Li, Tao Liao, Guo-Xin Ni

**Affiliations:** 1 Department of Rehabilitation Medicine, The First Affiliated Hospital of Fujian Medical University, Fuzhou, China; 2 School of Sport Medicine and Rehabilitation, Beijing Sport University, Beijing, China

## Abstract

**Objective:**

To perform a meta-analysis comparing the structural progression and clinical symptom outcomes as well as adverse events experienced from intra-articular injections of sprifermin compared to a placebo treatment for patients with knee osteoarthritis (KOA).

**Method:**

We systematically searched the literature for studies that compared long-term outcomes between sprifermin and placebo injections for KOA treatment. Meta-analysis was performed with RevMan5.3 using an inverse variance approach with fixed or random effects models. Odds ratios (ORs) and 95% confidence intervals (CIs) were estimated.

**Results:**

Eight studies were included. Overall, there was significantly less improvement of WOMAC total scores in patients receiving sprifermin, compared with the placebo (mean difference (MD) = 3.23, 95% CI 0.76–5.69; *I*^2^ = 0%; *P* = 0.01). Further, sprifermin injection patients gained more, and lost less, cartilage thickness and volume in total femorotibial joint (cartilage thickness: standardized mean differences (SMD) = 0.55, 95% CI 0.26–0.84; *I*^2^ = 78%; *P* = 0.0002; cartilage volume: SMD = 0.39, 95% CI 0.20–0.58; *I*^2^ = 49%; *P* < 0.0001). Changes in the cartilage surface morphology of the medial tibio-femoral joint (MD = −0.30, 95% CI −0.44 to −0.16; *I*^2^ = 0%; *P* < 0.0001) and patello-femoral joint (MD = −0.22; 95% CI −0.37 to −0.07; *I*^2^ = 0%; *P* = 0.004) showed a significant difference between the sprifermin and placebo injections. Moreover, there were no significant differences between sprifermin and the placebo in the risk of treatment-emergent adverse events (OR = 1.05; 95% CI 0.52–2.14; *I*^2^ = 48%; *P* = 0.89).

**Conclusion:**

The data from the included studies provide strong evidence to determine the effect of intra-articular sprifermin on joint structure in individuals with KOA and show no specific adverse effects. Nevertheless, intra-articular sprifermin did not likely have any positive effect on symptom alleviation.

## Key messages

- Intra-articular sprifermin is safe for the management of knee osteoarthritis.
- Intra-articular sprifermin may result in an improvement in cartilage thickness, volume, and surface morphology in KOA patients.
- Intra-articular sprifermin does not likely have any positive effect on symptom alleviation.

## Introduction

Osteoarthritis (OA) is the most common type of arthritis and a leading cause of mobility-related disability [1]. It affects about 10% of those around the world who are 60 years and over and causes substantial economic burdens and socioeconomic consequences [1, 2]. The symptoms of knee osteoarthritis (KOA), the knee being one of the most commonly affected joints, include limited range of motion, joint swelling, and pain that can cause disability [3].

Current therapies for OA are largely palliative and mainly focus on alleviating symptoms [4]. There are no pharmacological treatments known to prevent or cure OA [5]. International guidelines recommend the use of topical non-steroidal anti-inflammatory drugs (NSAIDs) and/or paracetamol as the first-line treatment of choice [3]. These drugs may relieve pain, but their prolonged consumption can result in severe adverse effects [3, 6]. Similarly, intra-articular injection of agents such as corticosteroids and hyaluronic acid may alleviate symptoms in KOA patients [7], but their effects on long-term clinical outcomes seem to be controversial [3, 7]. Given the nature of this chronic disorder, lifelong treatment should be required to arrest or slow its progression. Consequently, there is an urgent need for disease-modifying therapies that can alleviate symptoms and be safe for clinical use over long periods of time [8].

Sprifermin, a recombinant human fibroblast growth factor 18, is currently being investigated as a potential disease-modifying OA drug [9]. Evidence has been accumulating to indicate that it could stimulate chondrocyte proliferation and increase knee joint cartilage thickness [10, 11]. Recently, several placebo-controlled clinical trials [12, 13] have been conducted. While beneficial effects on the knee joint structure and clinical symptoms in KOA patients were reported in some studies [13, 14], others saw inconsistent results [12, 14]. As such, an update on the current evidence is required. A meta-analysis was performed in this study to provide a much-needed and comprehensive assessment on the efficacy and safety of sprifermin injection and to evaluate its potential for clinical application in KOA patients.

## Methods

The study protocol was registered with the International Prospective Register of Systematic Reviews (PROSPERO) (number CRD42020184508).

### Literature search

A search of studies published between the start of each database and September 2020 was conducted using bibliographic databases, including PubMed, Embase, the Cochrane Library, and Ovid. We used a series of keyword combinations (Medical Subject Headings [MeSH]) and text terms in the titles and abstracts that described OA and sprifermin injection (full search strategy available in Supplement 1).

### Eligibility criteria

Papers that met the following criteria were included in the analysis: (1) study design—RCT studies; (2) participants—patients with symptomatic knee OA at a Kellgren-Lawrence grade ≧ 2; (3) grouping—in addition to a therapy group with sprifermin injections, a control group receiving placebo treatments was included; (4) outcomes—outcomes reflecting efficacy (including symptoms, physical function, knee structure) and safety; and (5) language—published in English language journals.

The following were the exclusion criteria: (1) experimental studies (e.g., animal studies), (2) studies where the follow-up time is less than 1 month, (3) studies where sprifermin was combined with other drugs, (4) studies whose full text is not available, and (5) studies with no data available.

### Study selection and data extraction

Two researchers (NZ and XYC) independently reviewed all the retrieved abstracts and full texts. If any disagreement was raised, it was resolved through discussion and consultation with a third researcher (ZPY). The following data were separately extracted by the two reviewers from the included studies: publication year, study design, number of participants, comparison group, treatment protocol of sprifermin, and outcome measures (such as the Western Ontario and McMaster Universities Osteoarthritis Index [WOMAC]), pain scores, cartilage thickness and morphology, treatment-emergent adverse events (TEAEs), and acute inflammatory reactions (AIRs).

### Risk of bias assessment

Two researchers (NZ and XYC) independently evaluated the risk of bias in the included studies, using the Cochrane handbook [15, 16]. Seven domains were evaluated: generation of randomization sequences, allocation concealment, blinding of participants and implementers, blinding of outcome assessment, incomplete outcome data, selective reporting, and other potential biases. The assessment of each domain was classified as low, high, or unknown risk of bias. For any disagreements, a third consultant (ZPY) was available to resolve the matter and elicit consensus. Studies involving three or more high risks of bias in the seven domains were considered to possess poor methodological quality.

### Statistical analysis

Extracted data were analyzed using Review Manager V5.3. Continuous outcomes such as WOMAC pain scores, cartilage thickness, and cartilage volume were calculated and expressed as a weighted mean difference (WMD) (MD in RevMan V5.3) or as a standardized MD (SMD), while dichotomous data were expressed as an odds ratio (OR). A random-effects meta-analysis was used to compute a summary estimate, and a 95% CI was calculated for pooled estimates for each outcome. Statistical significance was considered to be *P* < 0.05. To assess heterogeneity between studies, *Q* and *I*^2^ statistics were calculated (*P* value less than 0.10 of the *Q* statistics indicates heterogeneity, and a value of less than 50% of the *I*^2^ statistics indicates low homogeneity, with a value of 75% or more indicating high heterogeneity) [17].

## Results

### Search results

**Fig. 1:**
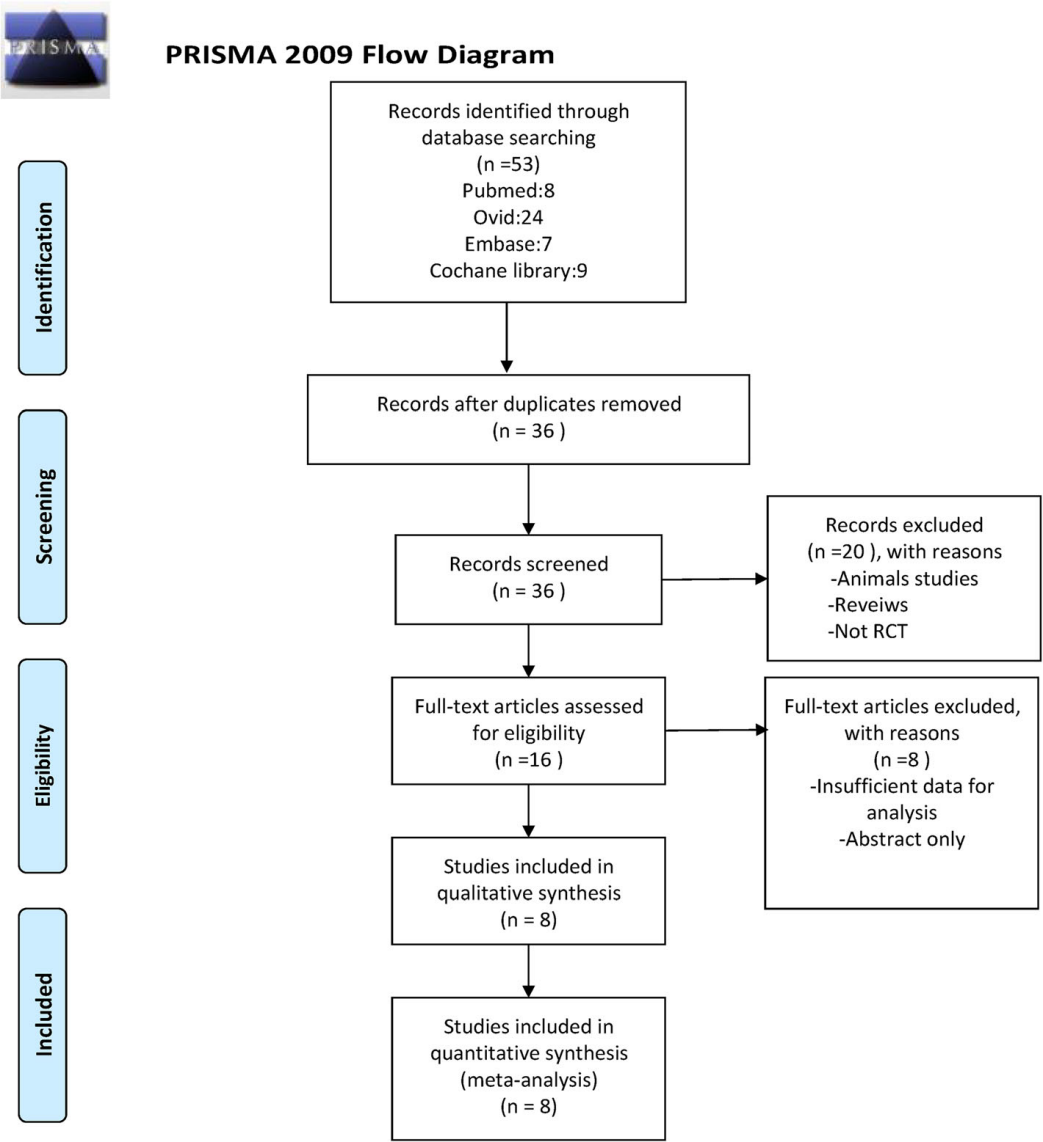
Flow chart of the study’s screening process

Database searches initially identified 53 studies. After removing duplicates, 36 studies were reviewed by title and abstract. Sixteen papers were screened in full text, with eight papers meeting the eligibility criteria. The PRISMA flow chart for study screening at each step was established, as shown in Fig. 1.

### Study characteristics and quality assessment

Characteristics of the included studies are listed in Table 1.

**Table 1:** Characteristics of studies included in the meta-analysis

Author (year)	Design	Number of patients	Sprifermin treatment strategy	Comparison	Follow-up	Outcomes
Lohmander 2014 [18]	RCT	*n* = 192	SAD/MAD Doses: 10, 30, 100 μg	Placebo	12 months	1, 2, 5, 6, 8, 11
Eckstein 2015 [19]	RCT	*n* = 168	Injected 3 times over 3 weeks Doses: 10, 30, 100 μg	Placebo	12 months	6, 7
Roemer 2020 [13]	RCT	*n* = 75	SAD/MAD Doses: 10, 30, 100 μg	Placebo	12 months	9, 10
Dahlberg 2016 [20]	RCT	*n* = 73	SAD/MAD Doses: SAD: 3, 10, 30, 100 μg; MAD: 3, 10, 30, 100, 300 μg	Placebo	6 months	1, 2, 3, 4
Roemer 2018 [21]	RCT	*n* = 549	100 μg q6mo, 100 μg q12mo, 30 μg q6mo, 30 μg q12mo	Placebo	6, 12, 18, 24 months	9, 10
Conaghan 2019 [22]	RCT	*n* = 549	100 μg q6mo, 100 μg q12mo, 30 μg q6mo, 30 μg q12mo	Placebo	2 years	6
Hochberg 2019 [12]	RCT	*n* = 549	100 μg q6mo, 100 μg q12mo, 30 μg q6mo, 30 μg q12mo	Placebo	2 years/3 years	1, 2, 3, 4, 5, 6, 8, 12
Eckstein 2020 [23]	RCT	*n* = 516	100 μg q6mo, 100 μg q12mo, 30 μg q6mo, 30 μg q12mo	Placebo	24 months	6, 7

Outcomes: 1. treatment-emergent adverse events (TEAEs), 2. local TEAEs, 3. systemic TEAEs, 4. acute inflammatory reaction (AIRs), 5. Western Ontario and McMaster Universities Osteoarthritis Index (WOMAC), 6. cartilage thickness, 7. ordered values (OVs), 8. cartilage volume, 9. cartilage morphology, 10. bone marrow lesions (BMLs), 11. joint space width (JSW), and 12. minimum joint space width (mJSW)

*SAD* single ascending dose, *MAD* multiple ascending dose, *q6mo and q12mo* sprifermin administered every 6 months and every 12 months, respectively

**Fig. 2:**
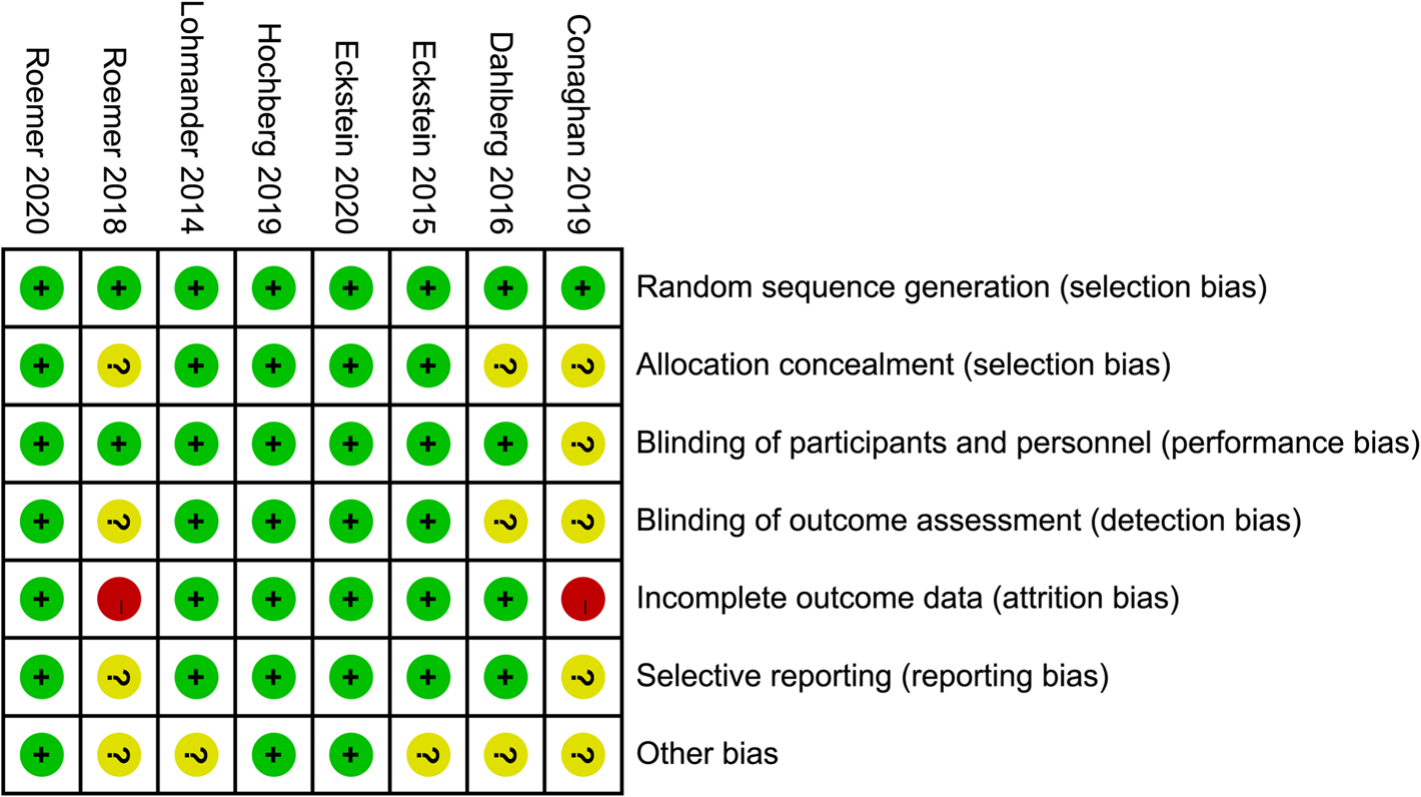
Quality assessment of the included studies. The green background with “+” means low risk of bias; the red background with “−” means high risk of bias; the yellow background with “?” means unknown risk of bias

A total of eight studies were included in this meta-analysis [12, 13, 21], all published between 2014 and 2020, while there were only three original trials (NCT00911469 [Dahlberg 2016]; NCT01033994 [Lohmander 2014; Eckstein 2015; Roemer 2018]; NCT01919164 [Conaghan 2019; Hochberg 2019; Roemer 2020; Eckstein 2020]); the included studies analyzed different outcomes conducted on the three trials. All trials were comparison studies, including sprifermin and placebo, with a follow-up period of ≧6 months. The overall bias of the included studies is shown in Fig. 2. Most studies were rated with a “moderate risk of bias.”

### Efficacy of sprifermin injection for OA treatment

#### Symptoms change measurements

##### Change in WOMAC score from baseline

**Fig. 3:**
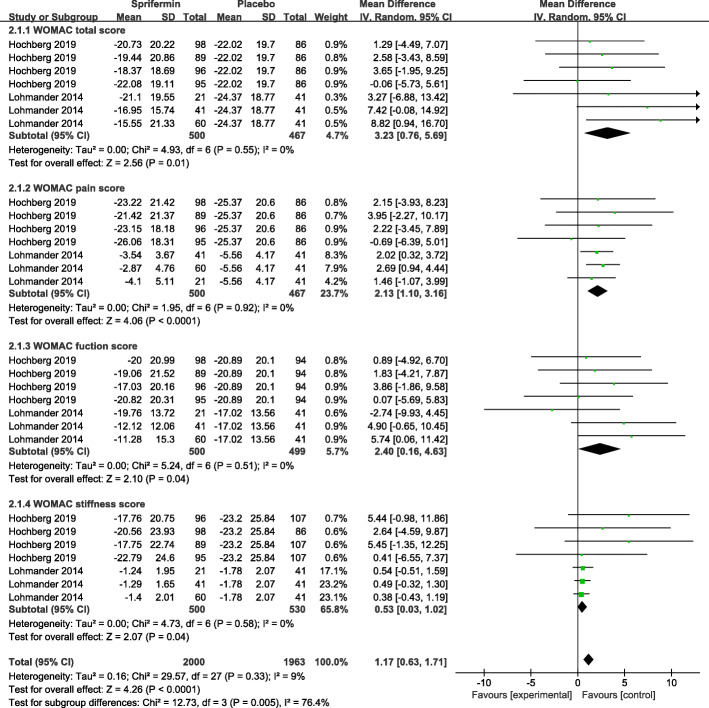
Effectiveness for sprifermin versus placebo in symptom alleviation. Forest plots of the mean difference (MD) with 95% confidence interval (CI) in WOMAC score (WOMAC total score, WOMAC pain score, WOMAC function score, WOMAC stiffness score) between patients undergoing sprifermin injection and placebo injection. (The three or four effect sizes for each trial in the figure represent the different dose of sprifermin treatment in the same trials)

Two studies (participants: sprifermin, *n* = 500; Placebo, *n* = 127) investigated the effect of sprifermin injection on symptom efficacy in KOA patients [12, 18], with Fig. 3 showing the outcomes of the meta-analysis for clinical symptoms and joint function. Symptom efficacy was evaluated as the change from baseline at 12 months [18] to 3 years [12] using WOMAC score. In comparison with the placebo group, sprifermin recipients exhibited less improvement in WOMAC scores, including for total (MD = 3.23, 95% CI 0.76–5.69; *I*^2^ = 0%; *P* = 0.01), pain (MD = 2.13, 95% CI 1.10–3.16; *I*^2^ = 0%; *P* < 0.0001), function (MD = 2.40, 95% CI 0.16–4.63; *I*^2^ = 0%; *P* = 0.04), and stiffness (MD = 0.53, 95% CI 0.03–1.02; *I*^2^ = 0%; *P* = 0.04).

#### Structural change measurements

##### Change in cartilage from baseline

1. Cartilage thickness

**Fig. 4:**
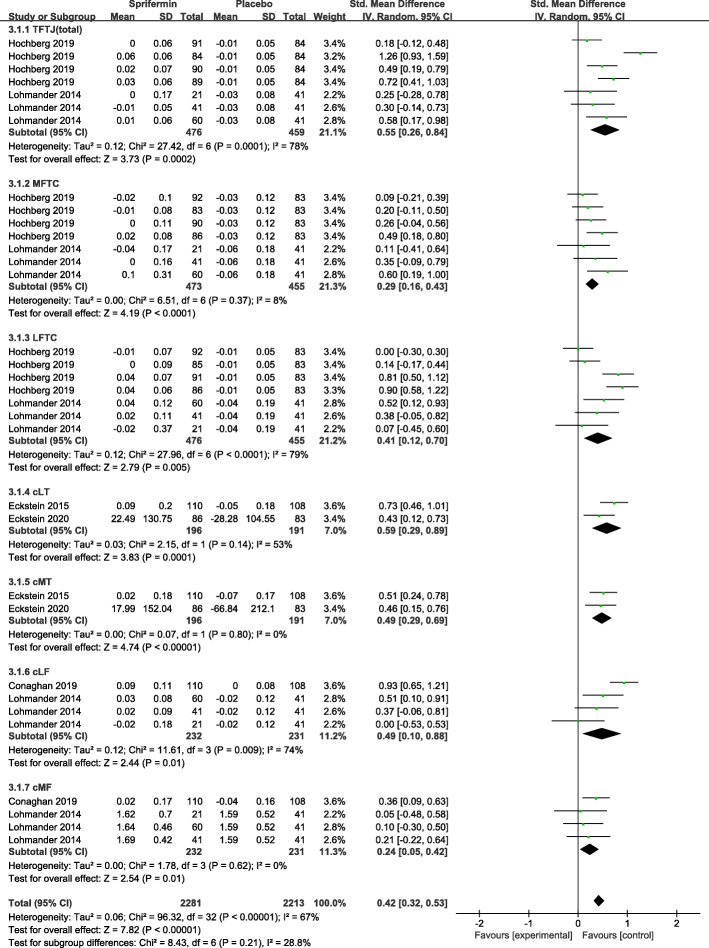
Effectiveness for sprifermin versus placebo in cartilage thickness improvement. Forest plots of standardized mean difference (SMD) with 95% confidence interval (CI) in total cartilage thickness and cartilage thickness in femorotibial subregional (MFTC, LFTC, cMT, cLT, cMF, cLF) between patients undergoing sprifermin injection and placebo injection. (The three or four effect sizes for each trial in the figure represent the different dose of sprifermin treatment in the same trials)

Five RCTs were included for the meta-analysis of cartilage thickness management [12, 18–20, 22, 23]. Figure 3 shows a significant difference in the change from baseline in the total femorotibial joint (TFTJ) cartilage thickness of the experimental group (participants: *n* = 476) compared with the control group (participants: *n* = 125) (SMD = 0.55, 95% CI 0.26–0.84; *I*^2^ = 78%; *P* = 0.0002). Sprifermin-treated patients gained more, and subsequently lost less, cartilage thickness in the femorotibial subregions (lateral femorotibial compartments (LFTC), medial femorotibial compartment (MFTC), cMT, cLT, cMF, cLF) versus placebo-treated patients (Fig. 4). Also, five of 16 location-independent ordered values (OVs) of subregional change in cartilage thickness were analyzed, with the difference amounting to a statistical significance in all OVs assessed (MD = 45.43, 95% CI 36.69–54.18; *I*^2^ = 0%; *P* < 0.00001) (eFigure 1 in Supplement 2).

2. Cartilage volume

Two studies (participants: sprifermin, *n* = 473; Placebo, *n* = 124) were included for meta-analysis of cartilage volume change [12, 18]. As shown in eFigure 2 in Supplement 2, sprifermin injection led to higher rates of improvement of cartilage volume in the total knee region (SMD = 0.39, 95% CI 0.20–0.58; *I*^2^ = 49%; *P* < 0.0001) and LFTC (SMD = 0.44, 95% CI 0.17–0.71; *I*^2^ = 75%; *P* = 0.001), but not in MFTC (SMD:0.11, 95% CI −0.11–0.32; *I*^2^ = 64%; *P* = 0.34) when compared with placebo injection.

3. Cartilage morphology

Two studies (participants: sprifermin, *n* = 801; placebo, *n* = 189) evaluated the cartilage surface morphology using magnetic resonance images (MRI) [13, 21]. According to the results of the meta-analyses, cartilage surface morphology saw improvement in sprifermin-treated knees compared to placebo-treated knees, with effects being significant in the PFJ (MD = −0.22, 95% CI −0.33to −0.11; *I*^2^ = 0%; *P* = 0.0001) and medial tibio-femoral joint (MFTJ) (MD = −0.30, 95% CI −0.44 to −0.16; *I*^2^ = 0%; *P* < 0.0001), but not in the whole knee (*P* = 0.06) and lateral tibio-femoral joint (LFTJ) (*P* = 0.35) (eFigure 3 in Supplement 2).

##### Change in BMLs from baseline

Two studies (participants: sprifermin, *n* = 800; placebo, *n* = 190) reported on bone marrow lesions (BMLs) using MRI [13, 21]. The application of sprifermin injection has no significant impact on the change of BMLs (included the whole knee and LTFJ, MTFJ, and PFJ) (eFigure 4 in Supplement 2).

##### Change in joint space width

Two studies (participants: sprifermin, *n* = 517; placebo, *n* = 128) reported on joint space width (JSW) from radiographs [12, 18]. Sprifermin was associated with statistically significant JSW narrowing in the lateral femorotibial compartment (MD 0.19, 95% CI 0.09–0.30; *I*^2^ = 44%; *P* = 0.0002) (eFigure 5 in Supplement 2).

#### Safety of sprifermin injections for osteoarthritis treatment

##### Adverse events

**Fig. 5:**
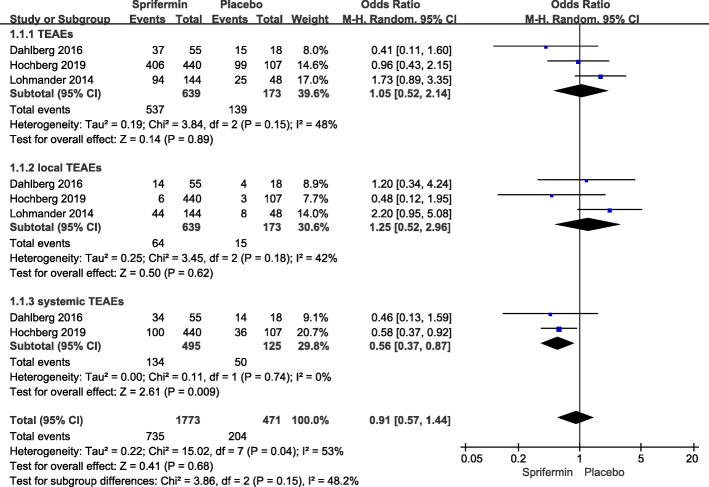
Safety for intra-articular sprifermin versus placebo. Forest plots of odds ratio (OR) with 95% confidence interval (CI) in TEAEs, local TEAEs, and systemic TEAEs between patients undergoing sprifermin injection and placebo injection

Three studies (participants: sprifermin, *n* = 639; placebo, *n* = 173) assessed the risk of overall and local TEAEs [12, 18, 20], and two (participants: sprifermin, *n* = 495; placebo, *n* = 125) assessed the risk of systemic TEAEs [12, 20]. While the significantly decreased risk of systemic TEAEs was revealed with sprifermin injection (OR = 0.56, 95% CI 0.37–0.87; *I*^2^ = 0%; *P* = 0.009), no significant differences were found in the risk of overall TEAEs (OR = 1.05, 95% CI 0.52–2.14; *I*^2^ = 48%; *P* = 0.89) or in the risk of local TEAEs (OR = 1.25, 95% CI 0.52–2.96; *I*^2^ = 42%; *P* = 0.62) or between sprifermin and placebo injections, respectively (Fig. 5).

##### Acute inflammatory reaction

Two studies (participants: sprifermin, *n* = 495; placebo, *n* = 125) assessed the risk of acute inflammatory reaction (AIR) [12, 20]. There was no statistical difference in the risk of acute inflammatory reaction (AIR) between the sprifermin and placebo injection groups (OR = 1.71, 95% CI 0.95–3.07; *I*^2^ = 0%; *P* = 0.07) (eFigure 6 in Supplement 2).

## Discussion

To the best of our knowledge, this is the first meta-analysis that aims to provide a comprehensive evaluation of the efficacy and safety of intra-articular sprifermin injections in KOA patients. The data from the included studies provided sufficient evidence to determine no specific adverse effects on the joint structure in individuals with KOA receiving intra-articular sprifermin injections. Nevertheless, its effect on symptom alleviation seems to be inconclusive.

Sprifermin is a potential disease-modifying OA drug for KOA [3, 9]. Previous in vitro studies suggested its enhancement in chondrocyte proliferation and overall extracellular matrix production, as well as an increased repair response of mechanically damaged articular cartilage [10, 24]. The data in the included RCT studies demonstrated that sprifermin has a beneficial effect on cartilage thickness, volume, and morphology in KOA patients, with the effect appearing to be location-specific. A preferential effect was revealed on either the lateral knee compartment or the medial compartment of the knee joint [18, 19, 23]. Since clinical trials may not account for clear differences in disease laterality, location-dependent effects on the cartilage would need further investigations [20]. However, it is worthwhile to note that dosage may be a crucial factor. Hochberg et al. reported that, in comparison with placebo, intra-articular sprifermin with 100 μg of every 6 or 12 months induced a significant improvement in total femorotibial joint cartilage thickness, while intra-articular sprifermin with 30 μg failed to induce a significant improvement [12]. On the other hand, in terms of BMLs, significant improvement was not found in the intra-articular sprifermin meta-analysis. The effects of sprifermin on subchondral BMLs, patterns that emerge from MRIs of the knees with OA and relate to structural and symptomatic progression of OA, seem to largely depend on the duration, since the improvement of BMLs was observed at a 24-month follow-up [21], but not at 6 or 12 months [14, 21]. Further investigations are warranted to better understand the effects of intra-articular sprifermin on the improvement of BMLs.

Our data demonstrated that it is unlikely that intra-articular sprifermin leads to significant improvement in physical function and clinical symptoms in KOA patients. However, this finding should be taken with a grain of salt since the effects seem to change with time and in relation to patient baseline characteristics. When compared to the placebo, intra-articular sprifermin led to significantly less improvement at year 1 [18] with no statistical differences at years 2 and 3 [12], as well as a significantly greater improvement at year 3 in the “at-risk” subgroup (a patient subgroup with higher pain scores and lower joint space width at baseline) [25] among the WOMAC total scores. Further investigations are required to understand its long-term effect on clinical symptoms and functions in a targeted population. Additionally, even though DMOAD may not prevent pain in the latter stages of OA [18], its effect in preserving cartilage may prolong the time before KOA patients reach levels of debilitating pain [12, 18].

In addition to its efficacy, the safety of intra-articular sprifermin was also demonstrated in this study, suggesting that sprifermin holds potential for clinical application in KOA patients. Another important aspect to consider when interpreting the results is the study quality. Two included studies [21, 22] are conference abstracts, which makes it difficult to assess their quality. And Dahlberg et al. [20] only reported that participants were randomized but gave no explanation of the procedure and did not report the method used to conceal the allocation sequence in sufficient detail. All studies presented performance bias due to the impossibility of blinding personnel and participants. For example, interactive web/voice response systems were used to assign a blinded treatment kit number to each participant at each visit for the administration of sprifermin in the original trials (NCT01033994 and NCT01919164).

Nevertheless, this study has several limitations. First, only eight studies from three original trials were included in this study. As such, several key indicators were not analyzed in this study, like the visual analog scale. Another limitation relates to the heterogeneity of the outcome. Although statistical heterogeneity does not exist in most outcomes among the included studies (*I*^2^ < 50%), significant heterogeneity does exist in a couple of outcomes. For example, total cartilage thickness has an *I*^2^ of 78%. Subgroup analysis was not performed due to the limited articles included, which appears to compromise our findings. However, we suggested that the high level of heterogeneity among various studies is due to different treatment protocols (such as intra-articular dosage). When evaluating the change from baseline in total cartilage thickness of the sprifermin group compared with the control group, by including the outcomes of sprifermin with the same dosage (like 100 μg) instead of all treatment dosage of sprifermin, *I*^2^ was changed from 78 to 0%. Furthermore, most included studies were rated with “moderate risk of bias.” The quality of these trials may reduce the confidence in the effect estimates observed in the present meta-analyses. More high-quality RCT trials are required to better understand the efficacy and safety of intra-articular sprifermin in the treatment of KOA patients.

## Conclusion

The data from the included studies provides strong evidence to determine the effect of intra-articular sprifermin on the joint structure in individuals with KOA and shows no specific adverse effects. On the other hand, intra-articular sprifermin did not likely have any positive effect on symptom alleviation. While sprifermin can be regarded as a potential DMOAD for KOA patients, more evidence is still required for its efficacy and safety.

## Supplementary Information

**Additional file 1.** Search strategies for Pubmed, EMBASE, The Cochrane Library database and Ovid.

**Additional file 2.** Forest plots of intra-articular Sprifermin vs placebo on OVs, cartilage volume, cartilage morphology, BMLs, JSW and AIRs. **Figure 1.** Forest plots of mean difference (MD) with 95% confidence interval (CI)in OVs between patients undergoing Sprifermin injection and placebo injection. **Figure 2.** Forest plots of standardised mean difference (SMD) with 95% confidence interval (CI) in total cartilage volume and cartilage volume in femorotibial subregional (MFTC, LFTC) between patients undergoing Sprifermin injection and placebo injection. (The three or four effect sizes for each trial in the figure represents different dose of Sprifermin treatment in the same trials). **Figure 3.** Forest plots of mean difference (MD) with 95% confidence interval (CI) in cartilage morphology of whole knee, MTFJ, LTFJ and PFJ between patients undergoing Sprifermin injection and placebo injection. (The three or four effect sizes for each trial in the figure represents different dose of Sprifermin treatment in the same trials). **Figure 4.** Forest plots of mean difference (MD) with 95% confidence interval (CI) in BMLs of whole knee, MTFJ, LTFJ and PFJ between patients undergoing Sprifermin injection and placebo injection. (The three or four effect sizes for each trial in the figure represents different dose of Sprifermin treatment in the same trials). **Figure 5.** Forest plots of mean difference (MD) with 95% confidence interval (CI) in JSW of medial and lateral femorotibial compartment between patients undergoing Sprifermin injection and placebo injection. (The three or four effect sizes for each trial in the figure represents different dose of Sprifermin treatment in the same trials). **Figure 6.** Forest plots of odds ratio (OR) with 95% confidence interval (CI) in AIRs, between patients undergoing Sprifermin injection and placebo injection.

## Data Availability

Not applicable.

## Abbreviations

AIRs: Acute inflammatory reactions
BMLs: Bone marrow lesions
CIs: Confidence intervals
JSW: Joint space width
KOA: Knee osteoarthritis
LFTC: Lateral femorotibial compartments
MD: Mean difference
MFTC: Medial femorotibial compartment
MeSH: Medical Subject Headings
MRI: Magnetic resonance images
mJSW: Minimum joint space width
NSAIDs: Non-steroidal anti-inflammatory drugs
ORs: Risk ratios
OVs: Ordered values
PFJ: Patello-femoral joint
RCTs: Randomized controlled trials
SMD: Standardized mean difference
TEAEs: Treatment-emergent adverse events
TFTJ: Total femorotibial joint
WOMAC: Western Ontario and McMaster Universities Osteoarthritis Index
