# Supplementary material for: Efficacy and safety of sprifermin injection for knee osteoarthritis treatment: a meta-analysis
Source: Arthritis Res Ther. 2021 Apr 9;23:107. doi: 10.1186/s13075-021-02488-w (PMC8034149; doi:10.1186/s13075-021-02488-w)
Supplement: Supplementary file 1 — Additional file 1. Search strategies for Pubmed, EMBASE, The Cochrane Library database and Ovid. [file 13075_2021_2488_MOESM1_ESM.doc]

**Supplementary 1**

**Search strategies for Pubmed, EMBASE, The Cochrane Library database and Ovid**

**Pubmed**

1. "Osteoarthritis"[Mesh]
2. Osteoarthritides[Title/Abstract]
3. Osteoarthrosis [Title/Abstract]
4. Osteoarthroses [Title/Abstract]
5. Arthritis, Degenerative [Title/Abstract]
6. Arthritides, Degenerative [Title/Abstract]
7. Degenerative Arthritides [Title/Abstract]
8. Degenerative Arthritis [Title/Abstract]
9. Osteoarthrosis Deformans [Title/Abstract]
10. 1 or 2 or 3 or 4 or 5 or 6 or 7 or 8 or 9 or 10
11. Sprifermin [Title/Abstract]
12. FGF-18[Title/Abstract]
13. fibroblast growth factor 18, human [Title/Abstract]
14. FGF-18 protein, human [Title/Abstract]
15. FGF18 protein, human [Title/Abstract]
16. 11 or 12 or 13 or 14 or 15 or 16
17. randomized controlled trial[Publication Type]
18. randomized[Title/Abstract]
19. placebo[Title/Abstract]
20. 17 or 18 or 19
21. 10 and 16 and 20

**EMBASE**

#1 ‘Sprifermin’/exp

#2 ‘FGF-18’:ab,ti

#3 ‘fibroblast growth factor 18, human’:ab,ti

#4 ‘FGF-18 protein, human’:ab,ti

#5 ‘FGF18 protein, human’:ab,ti

#6 #1 OR #2 OR #3 OR #4 OR #5

#7 ‘Osteoarthritis’/exp

#8 ‘Osteoarthritides’:ab,ti

#9 ‘Osteoarthrosis’:ab,ti

#10 ‘Osteoarthroses’:ab,ti

#11 ‘Arthritis, Degenerative’:ab,ti

#12 ‘Arthritides, Degenerative’:ab,ti

#13 ‘Degenerative arthritide’:ab,ti

#14 ‘Osteoarthrosis deformans’:ab,ti

#15 ‘Degenerative arthritis’:ab,ti

#16 #7 OR #8 OR #9 OR #10 OR #11 OR #12 OR #13 OR #14 OR #15

#17 ‘randomized controlled trial’/exp

#18 #6 AND #16 AND #17

**The Cochrane Library**

#1 MeSH:[Osteoarthritis, Knee] explode all trees

#2 Osteoarthritides:ti,ab,kw or Osteoarthrosis:ti,ab,kw or Osteoarthroses:ti,ab,kw

or Arthritis, Degenerative:ti,ab,kw or Arthritides, Degenerative:ti,ab,kw or Degenerative arthritides:ti,ab,kw or Degenerative arthritis:ti,ab,kw or Osteoarthrosis Defomans:ti,ab,kw (Word variations have been searched)

#3 #1 OR #2

#4 FGF-18:ti,ab,kw or sprifermin:ti,ab,kw or fibroblast growth factor 18,

human:ti,ab,kw or FGF-18 protein, human:ti,ab,kw or FGF18 protein,

human:ti,ab,kw

#5 #3 AND #2

**Ovid**

#1 Osteoarthritis:ti

#2 Osteoarthritides:ti

#3 Osteoarthrosis:ti

#4 Osteoarthroses:ti

#5 Arthritis, Degenerative:ti

#6 Arthritides, Degenerative:ti

#7 Degenerative arthritide:ti

#8 Osteoarthrosis deformans:ti

#9 Degenerative arthritis:ti

#10 #1 OR #2 OR #3 OR #4 OR #5 OR #6 OR #7 OR #8 OR #9

#11 Sprifermin:ti

#12 FGF-18:ti

#13 fibroblast growth factor 18, human:ti

#14 FGF-18 protein, human:ti

#15 FGF18 protein, human:ti

#16 #11 OR #12 OR #13 OR #14 OR #15

#17 randomized controlled trial:ab,ti

#18 randomized:ab,ti

#19 placebo:ab,ti

#20 #17 OR #18 OR #19

#21 #10 AND #16 AND #20
