# Supplementary material for: Efficacy and safety of sprifermin injection for knee osteoarthritis treatment: a meta-analysis
Source: Arthritis Res Ther. 2021 Apr 9;23:107. doi: 10.1186/s13075-021-02488-w (PMC8034149; doi:10.1186/s13075-021-02488-w)
Supplement: Supplementary file 2 — Additional file 2. Forest plots of intra-articular Sprifermin vs placebo on OVs, cartilage volume, cartilage morphology, BMLs, JSW and AIRs. Figure 1. Forest plots of mean difference (MD) with 95% confidence interval (CI)in OVs between patients undergoing Sprifermin injection and placebo injection. Figure 2. Forest plots of standardised mean difference (SMD) with 95% confidence interval (CI) in total cartilage volume and cartilage volume in femorotibial subregional (MFTC, LFTC) between patients undergoing Sprifermin injection and placebo injection. (The three or four effect sizes for each trial in the figure represents different dose of Sprifermin treatment in the same trials). Figure 3. Forest plots of mean difference (MD) with 95% confidence interval (CI) in cartilage morphology of whole knee, MTFJ, LTFJ and PFJ between patients undergoing Sprifermin injection and placebo injection. (The three or four effect sizes for each trial in the figure represents different dose of Sprifermin treatment in the same trials). Figure 4. Forest plots of mean difference (MD) with 95% confidence interval (CI) in BMLs of whole knee, MTFJ, LTFJ and PFJ between patients undergoing Sprifermin injection and placebo injection. (The three or four effect sizes for each trial in the figure represents different dose of Sprifermin treatment in the same trials). Figure 5. Forest plots of mean difference (MD) with 95% confidence interval (CI) in JSW of medial and lateral femorotibial compartment between patients undergoing Sprifermin injection and placebo injection. (The three or four effect sizes for each trial in the figure represents different dose of Sprifermin treatment in the same trials). Figure 6. Forest plots of odds ratio (OR) with 95% confidence interval (CI) in AIRs, between patients undergoing Sprifermin injection and placebo injection. [file 13075_2021_2488_MOESM2_ESM.doc]

**Supplementary 2**

**Forest plots of intra-articular Sprifermin vs placebo on OVs, cartilage volume,** **cartilage morphology, BMLs, JSW and AIRs.**


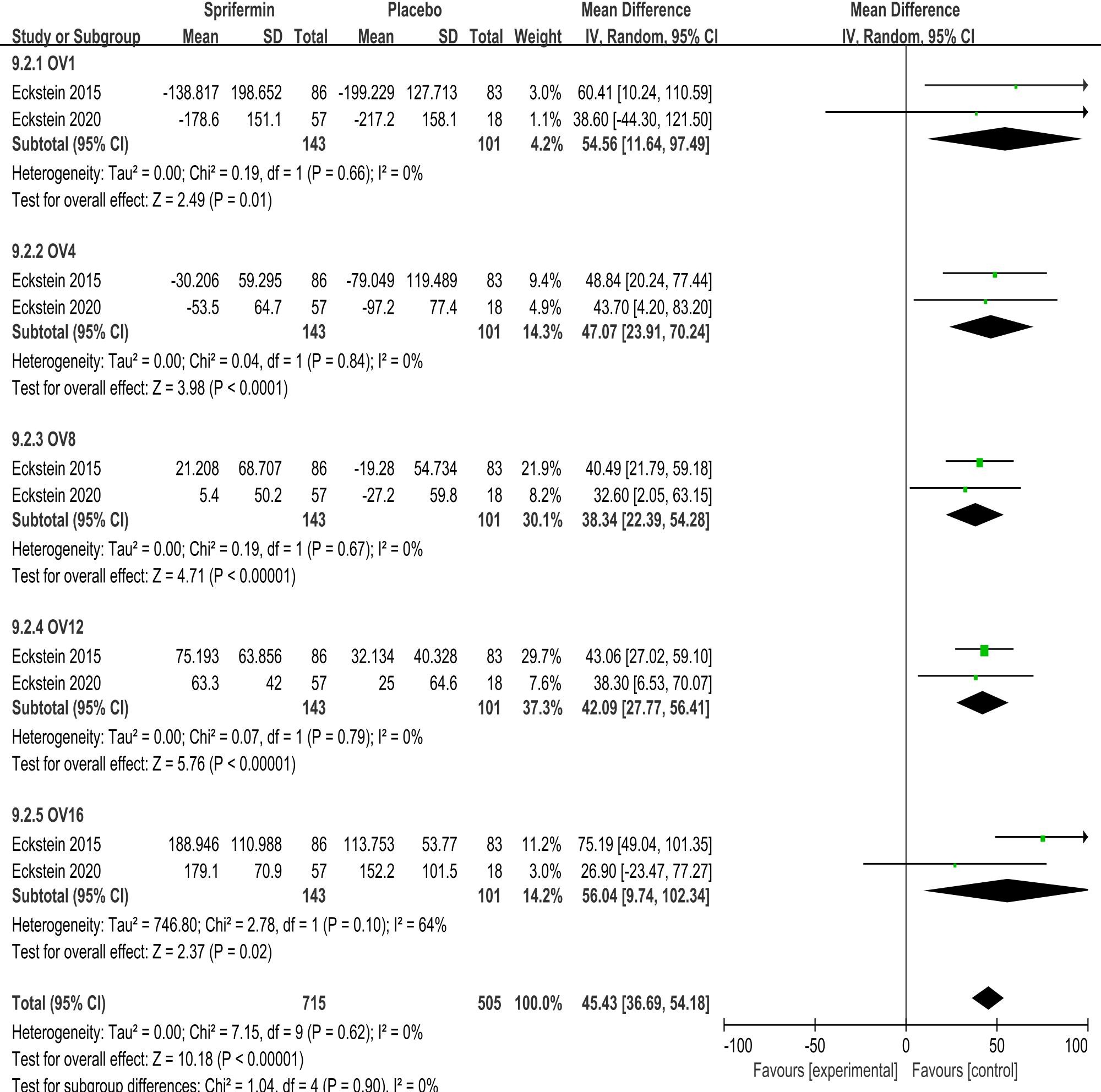


eFigure 1. Forest plots of mean difference (MD) with 95% confidence interval (CI)in OVs between patients undergoing Sprifermin injection and placebo injection.


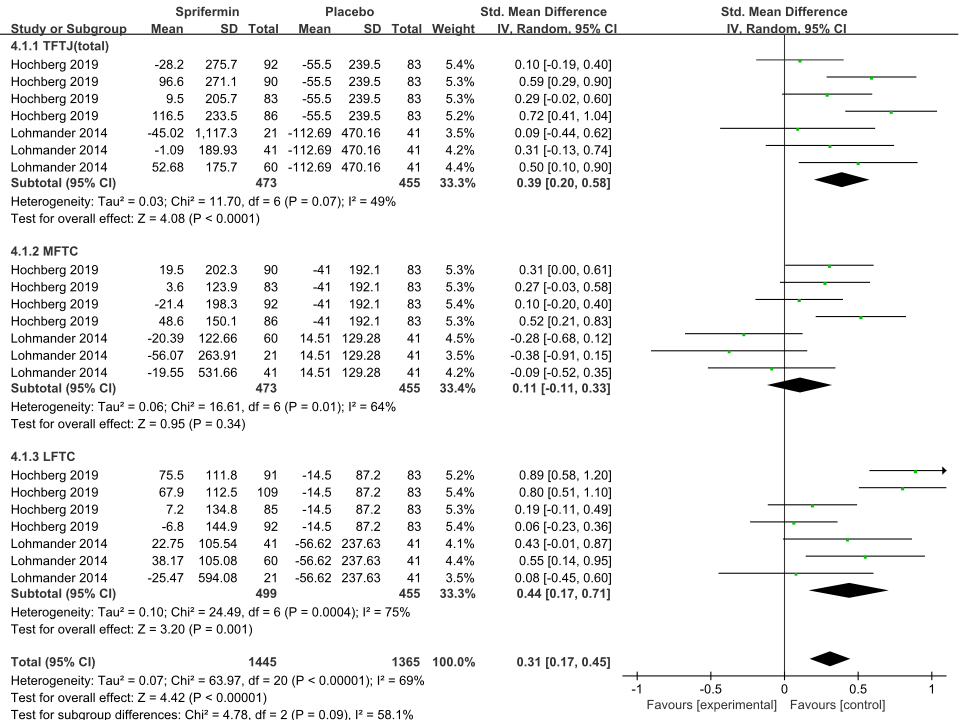


eFigure 2. Forest plots of standardised mean difference (SMD) with 95% confidence interval (CI) in total cartilage volume and cartilage volume in femorotibial subregional (MFTC, LFTC) between patients undergoing Sprifermin injection and placebo injection. (The three or four effect sizes for each trial in the figure represents different dose of Sprifermin treatment in the same trials.)


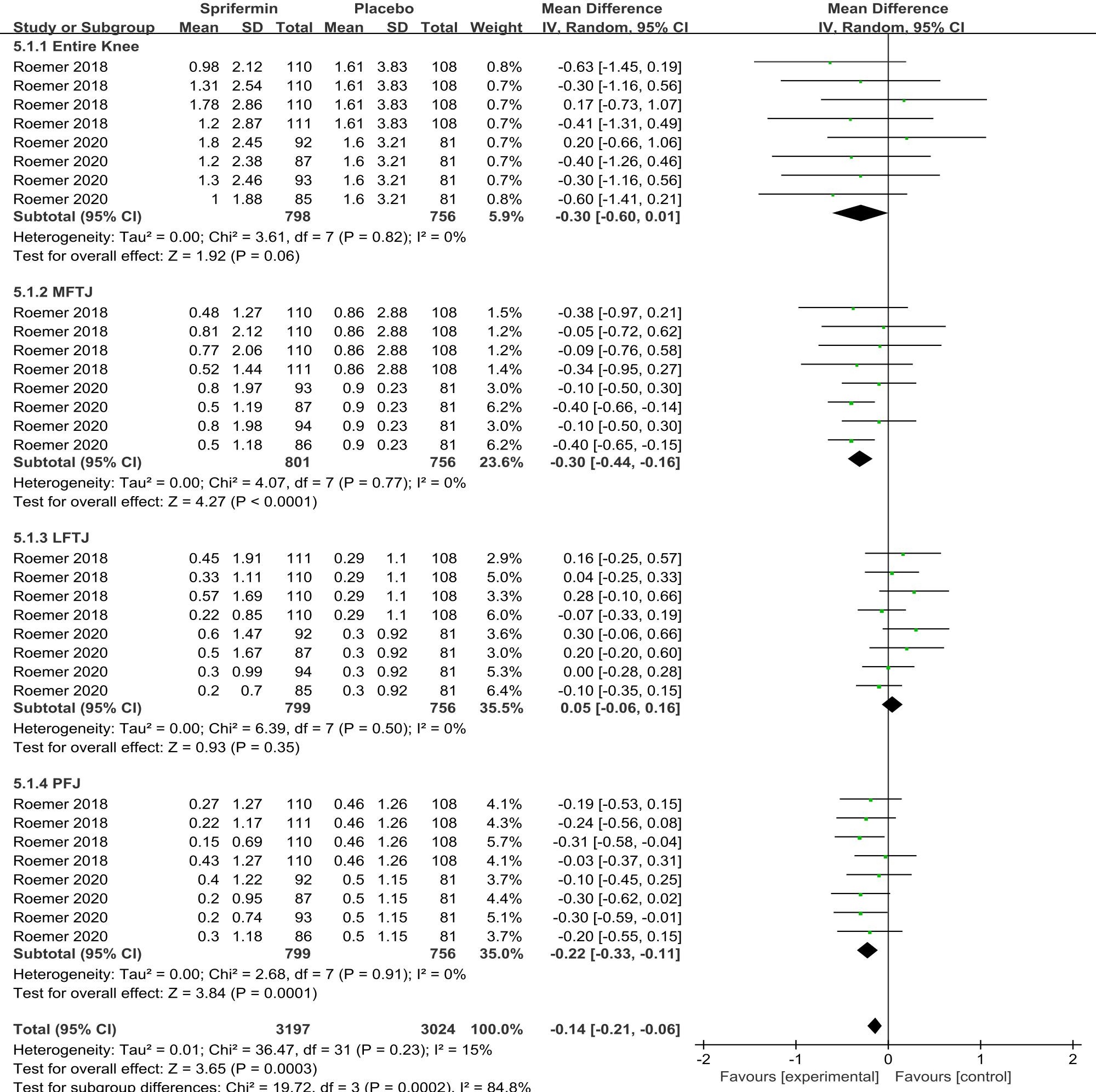


eFigure 3. Forest plots of mean difference (MD) with 95% confidence interval (CI) in cartilage morphology of whole knee, MTFJ, LTFJ and PFJ between patients undergoing Sprifermin injection and placebo injection. (The three or four effect sizes for each trial in the figure represents different dose of Sprifermin treatment in the same trials.)


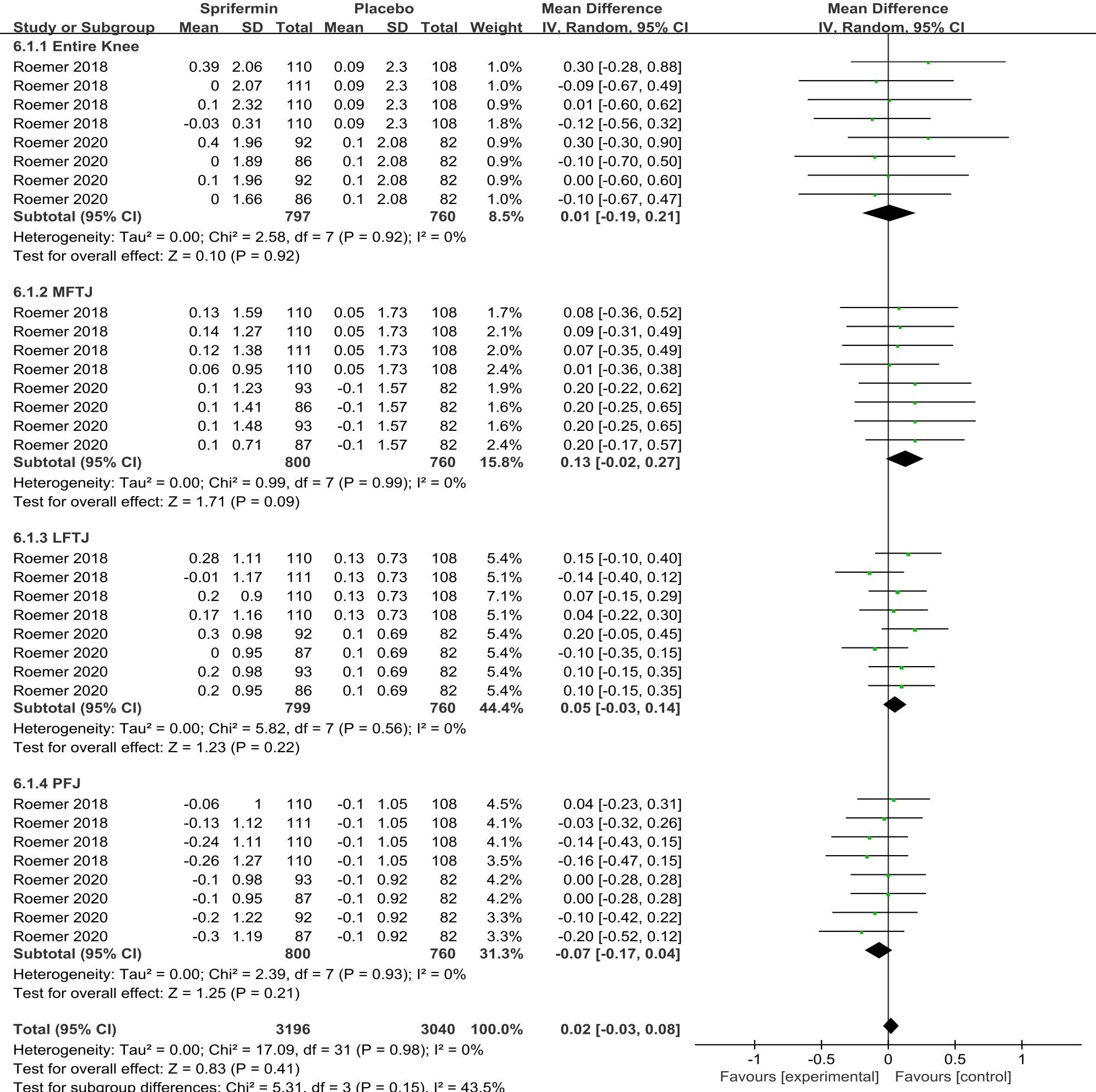


eFigure 4.Forest plots of mean difference (MD) with 95% confidence interval (CI) in BMLs of whole knee, MTFJ, LTFJ and PFJ between patients undergoing Sprifermin injection and placebo injection. (The three or four effect sizes for each trial in the figure represents different dose of Sprifermin treatment in the same trials.)


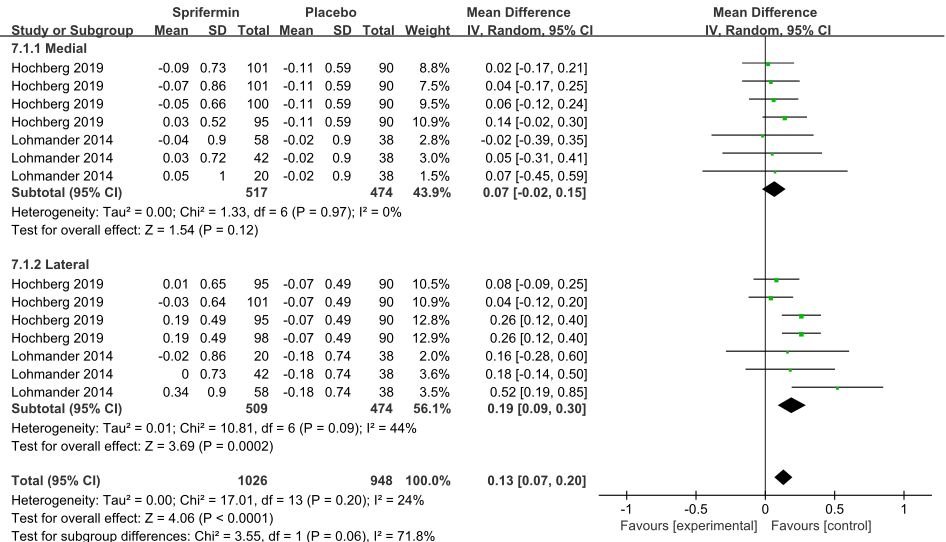


eFigure 5.Forest plots of mean difference (MD) with 95% confidence interval (CI) in JSW of medial and lateral femorotibial compartment between patients undergoing Sprifermin injection and placebo injection. (The three or four effect sizes for each trial in the figure represents different dose of Sprifermin treatment in the same trials.)


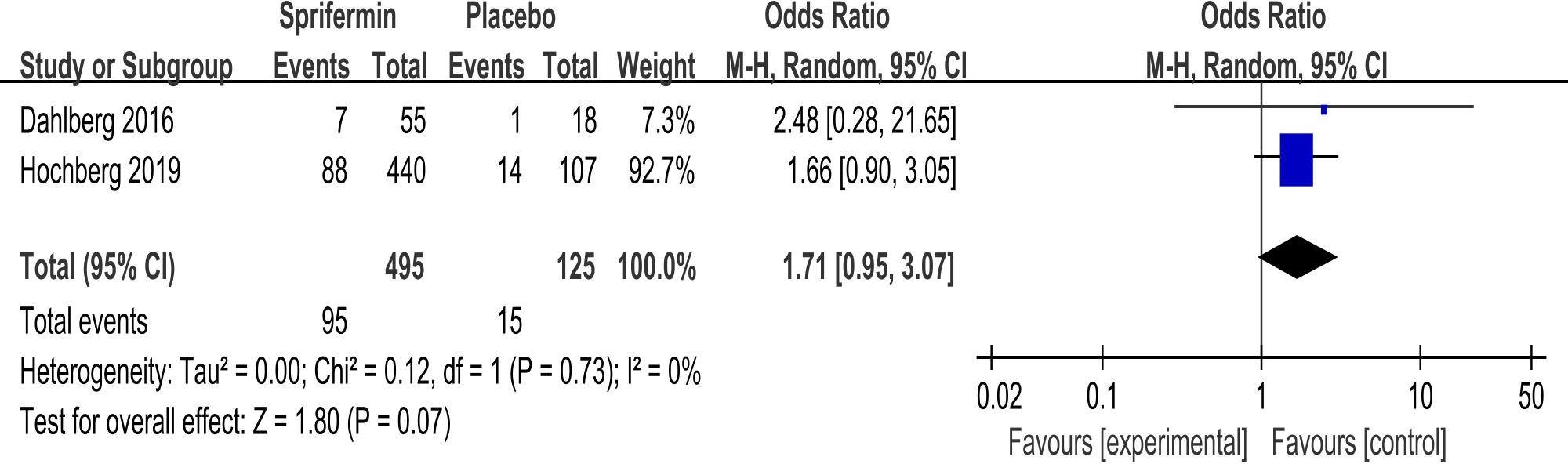


eFigure 6. Forest plots of odds ratio (OR) with 95% confidence interval (CI) in AIRs, between patients undergoing Sprifermin injection and placebo injection
